# Necrotizing fasciitis: modern clinical view

**DOI:** 10.1186/cc11747

**Published:** 2012-11-14

**Authors:** O Chernyshev, M Shatil, L Akinchits, N Vedernykova, V Kotlov, M Semak, N Bubnova

**Affiliations:** 1 City Hospital of St. George, Saint-Petersburg, Russia; 2 Saint-Petersburg State Medical University of I.P. Pavlov, Saint-Petersburg, Russia

## Background

Necrotizing fasciitis (NF) is one of the most severe diseases among surgical infections. There is always severe sepsis, multiple organ dysfunction, septic shock with NF and high mortality rates from 50% to 80 to 100%. Progressive severe sepsis with a lack of local inflammatory symptoms is being characterized as a modern clinical view of NF.

## Methods

A 5-year retrospective analysis of 12,907 admitted patients to the Department of Surgical Infections and Sepsis from 2007 until 2011 years was performed. All patients were admitted by the emergency service. The duration of admitting to the hospital from disease onset was 1 to 7 days, with a mean of 2 to 3 days.

## Results

There were 86 patients with NF in 2007 to 2011. All patients had severe sepsis or septic shock with multiple organ dysfunction proved by laboratory tests and were treated in the ICU. The most frequent accompanying immunodeficient disorders are diabetes, oncology, alcohol and drug addiction, and extensive contusion of soft tissues. There were six patients in 2007 with NF (2 discharged/4 died), 13 patients in 2008 (7/6), 18 patients in 2009 (6/12), 16 patients in 2010 (12/4), and 33 patients in 2011 (15/18) correspondingly. Obtained results show a stable increase of patients with NF. The amount of patients with NF by 2011 was extended 5.5 times compared with 2006. The mortality rates fluctuated between 55 and 66%, less mortality rate was seen in 2010 (25%). On average the mortality rate was 51.5%; this corresponds to the modern literature reviews. The most common organisms isolated were: *Enterococcus faecalis *(18.5%), *Staphylococcus aureus *(15.5%), Streptococcus (14%) and anaerobic cultures (6.6%). All admitted patients had been treated immediately with high doses of intravenous antibiotics and extensive surgical treatment of the infection lesions; following intensive treatment in the ICU was made. Serious attention to immunotherapy was made. Severe reduction of the lymphocytes and hemoglobin seemed clinically significant in prediction of inflammation severity.

## Conclusion

Accompanying immunodeficient disorders influence rising NF. Early diagnostics and active treatment make better treatment results and decrease mortality rates. Progressive decrease of lymphocytes and hemoglobin is extremely adverse to predict. Using immunotherapy makes clinical results much better. Regular microbiological monitoring of wound infection should make adequate antibiotic therapy.

